# Strain Echocardiography Is a Promising Tool for the Prognostic Assessment of Sarcoidosis

**DOI:** 10.3390/life11101065

**Published:** 2021-10-10

**Authors:** Paolo Cameli, Maria Concetta Pastore, Giulia Elena Mandoli, Mariangela Vigna, Giuseppe De Carli, Laura Bergantini, Miriana d’Alessandro, Nicolò Ghionzoli, Elena Bargagli, Matteo Cameli

**Affiliations:** 1 Respiratory Diseases Unit, Department of Medical Sciences, Siena University Hospital, 53100 Siena, Italy; 2 Department of Medical Biotechnologies, Section of Cardiology, University of Siena, 53100 Siena, Italy; pastore2411@gmail.com (M.C.P.); giulia_elena@hotmail.it (G.E.M.); mariangelavigna@gmail.com (M.V.); giuseppe.dcr93@yahoo.it (G.D.C.); nicologhionzoli@gmail.com (N.G.); matteo.cameli@yahoo.com (M.C.); 3 Respiratory Diseases Unit, Department of Medical and Surgical Sciences & Neurosciences, Siena University Hospital, 53100 Siena, Italy; laurabergantini@gmail.com (L.B.); dalessandro.miriana@gmail.com (M.d.); bargagli2@gmail.com (E.B.)

**Keywords:** sarcoidosis, biomarkers, echocardiography, strain

## Abstract

Sarcoidosis is a systemic chronic granulomatous disease with significant morbidity and mortality. Although basic transthoracic echocardiography (TTE) is not recommended for the assessment of sarcoidosis, speckle tracking echocardiography (STE) has emerged as more sensitive for the early detection of cardiac sarcoidosis and its outcome. The aim of the study was to assess the utility of left atrial and left ventricular longitudinal STE for the prediction of major adverse cardiac events (MACE) and sarcoidosis relapses. We enrolled 172 consecutive patients with sarcoidosis who underwent TTE and pulmonary function tests (PFTs). All patients were followed for a sarcoidosis relapse and MACE. During a median follow-up of 2217 days, 8 deaths, 23 MACE and 36 sarcoidosis relapses were observed. LV global longitudinal strain (GLS) was significantly lower in patients with MACE (*p* = 0.025). LV-GLS < 17.13% (absolute value) was identified as a fair predictor of MACE. Concerning the sarcoidosis control, TTE revealed a reduction of the LV ejection fraction (*p* = 0.0432), tricuspid annular plane systolic excursion (*p* = 0.0272) and global peak atrial longitudinal strain (PALS, *p* = 0.0012) in patients with relapses. PALS < 28.5% was the best predictor of a sarcoidosis relapse. Our results highlight a potential role of LV-GLS and PALS as prognostic markers in sarcoidosis, supporting the use of STE in the clinical management of these patients.

## 1. Introduction

Sarcoidosis is a chronic granulomatous disease with an unknown etiology associated with a significant morbidity, reduced quality of life and increased mortality [1,2]. The histopathological hallmark is sarcoid granuloma, which can potentially affect every organ of the body, determining the systemic nature of this disease. Although it is commonly defined and approached as a benign disease, the unpredictable clinical course and the potential involvement of vital organs make sarcoidosis a real challenge for clinicians. If not correctly assessed and treated, sarcoidosis may virtually determine an irreversible damage of the involved tissue, leading to organ failure and, not rarely, to exitus [3,4]. However, due to the wide heterogeneity of the disease severity and activity, a reliable prognostic assessment for patients affected by sarcoidosis is still lacking. Many biomarkers, as well as radiological classifications or functional respiratory parameters, have been linked to the activity of the disease and proposed for the prognostic estimation of sarcoidosis but none of them are currently validated and approved for routinary clinical practice [5,6,7,8,9,10,11]. This issue is further complicated by the wide spectrum of clinical manifestations of sarcoidosis that can fluctuate from an asymptomatic state to a chronically progressive disease staggered with acute and unexpected relapses. Moreover, symptoms and a subjective clinical status are often not strictly related to the number or severity of disease localizations, making the therapeutic management and response to the treatment more difficult to assess and standardize. 

A recent document from the American Thoracic Society (ATS) focused on the standardization of the diagnostic pathway and the detection of sarcoid localizations of the disease and proposed specific tools (including imaging, laboratory or endoscopic procedures) to be implemented in the clinical practice for this aim but none of them were mentioned to have a prognostic value [12]. Among these, transthoracic echocardiography (TTE) was not recommended for the diagnosis of cardiac sarcoidosis and cardiac magnetic resonance imaging (MRI) and cardiac positron emission tomography (PET) were suggested as first- and second-line procedures for diagnostic and prognostic information, respectively. However, as a comprehensive guideline, the ATS document focused on “traditional” TTE and, in particular, with the left ventricular (LV) ejection fraction (EF) estimation and wall motion abnormalities related to coronary artery diseases. In the last decade, a new imaging semi-automatic technique of speckle tracking echocardiography (STE) has proven to be more sensitive in the early detection of myocardial damage and less operator-sensitive than traditional TTE [13]. Not surprisingly, many studies have demonstrated that STE is significantly better than TTE in the early detection of myocardial damage or sarcoid involvement and also in predicting cardiac events in the follow-up of sarcoidosis patients [13,14,15,16]. However, scarce data are available on the potential utility of STE in the prognostic assessment of sarcoidosis and the prediction of a sarcoidosis relapse. 

The aim of our study was to investigate if this non-invasive and reproductive echocardiographic technique was able to predict worsening or flare and to evaluate its potential as a prognostic tool in a large cohort of sarcoidosis patients. 

## 2. Materials and Methods

### 2.1. Study Population

We retrospectively enrolled in the study patients affected with chronic sarcoidosis, diagnosed and followed at the Regional Referral Centre for Sarcoidosis and Interstitial Lung Diseases of Siena from January 2010 to October 2020. All diagnoses were made according to international guidelines through a multidisciplinary discussion including physicians experienced in the clinical management of interstitial lung diseases [12,17]. As key inclusion criteria for the study population, the following procedures had to be available for data collection:

- TTE, including STE parameters, performed at the Division of Cardiology of Siena University Hospital;
- A medical examination and pulmonary function tests (PFTs), including the diffusing capacity of the lung for carbon monoxide (DLCO) assessment, within three months of TTE;
- No acute event or treatment escalation must have occurred between the execution of the TTE and PFTs.

All patients with a malignant disease or a history of acute coronary syndromes within 3 months of the medical examination were excluded from the study.

At the baseline, all demographic, clinical, therapeutic, radiological, functional, immunological, laboratory and echocardiographic data were retrospectively collected from medical records and entered into an electronical database for the statistical analysis. The clinical status of the disease was assessed through the Clinical Outcome Status (COS) scale as endorsed by the World Association of Sarcoidosis and Other Granulomatous diseases (WASOG) [18].

Pulmonary and extrapulmonary localizations of the disease were using specific diagnostic procedures. 

The clinical phenotypes of sarcoidosis were retrospectively assessed according to GenPhenResA criteria (group 1: abdominal disease; group 2: ocular, central nervous system, cardiac, cutaneous (OCCC) disease; group 3: musculoskeletal and cutaneous disease; group 4: hilar lymph adenopathy and intrathoracic localizations; group 5: extrapulmonary disease) [19]. 

Thoracic radiological features (specifically, chest X-ray (CXR) and high-resolution computed tomography (HRCT)) as well as laboratory and immunological data were included in the database if performed within 3 months of the TTE execution. Chest X-rays were classified according to the Scadding criteria [20].

### 2.2. Study Design

All patients underwent a clinical and functional follow-up according to the management protocol of the Respiratory Diseases Unit of Siena University Hospital. The study population was stratified according to the disease phenotypes, radiological classification and clinical status, as described in the previous subsection. Sarcoidosis clinical assessments, mortality and major cardiovascular events (MACE) (cardiovascular death, hospitalizations for a cardiac cause, major arrhythmias (i.e., atrial fibrillation or sustained ventricular tachycardias and/or ventricular fibrillation)) were collected throughout from the inclusion in the study to 1 October 2020. 

The sarcoidosis status was classified as worsened if a therapeutic step-up (increase in or start of steroids and/or immunosuppressive drugs) was needed according to the advice of the respiratory physician and/or if evidence of organ damage due to sarcoidosis was identified through a clinical, radiological and laboratory assessment during the follow-up according the latest guidelines [12,13,14,15,16,17].

This study was designed as retrospective and was approved by our local ethics committee (C.E.A.V.S.E. Tuscany, Italy, Markerlung number 17431).

### 2.3. Basic and Advanced Echocardiography

An echocardiographic examination was performed according to the American Society of Echocardiography/European Association of Cardiovascular Imaging (ASE/EACVI) recommendations for the chamber quantification [21] using a high-quality ultrasound machine (Vivid E9; GE Medical System, Horten, Norway) with patients in the left lateral recumbent position. 

The LV wall thickness and diameters were measured in a parasternal long-axis view. The right ventricular diameters, RV fractional area change (RVFAC) and sphericity index were calculated using a standard apical 4-chamber view. LV EF and the LA volume and area were assessed using the biplane modified Simpson method from the apical 4- and 2-chamber views. The LV dimensions and LA volume were indexed to the body surface area obtaining an LV mass index and LA volume index (LAVI). From the 4-chamber view, the tricuspid annulus plane systolic excursion (TAPSE) was measured by M-mode; maximum early diastolic (E) and late diastolic (A) velocities were assessed by a transmitral pulsed wave doppler to calculate the E/A ratio. The peak systolic (S′), early diastolic (E′) and late diastolic (A′) annular velocities were then obtained by tissue doppler imaging and the E/E′ ratio was calculated and used as the index of the LV filling pressure. Valvular heart disease was quantified by bidimensional echocardiography according to ASE recommendations [22]. The systolic pulmonary artery pressure (sPAP) was estimated as the sum of the systolic transtricuspid pressure gradient and the right atrial pressure derived from the diameter and collapsibility of the inferior vena cava.

### 2.4. Speckle Tracking Echocardiography

An STE analysis was conducted on apical 2-, 3- and 4-chamber images obtained by 2D greyscale echocardiography with a stable electrocardiographic recording. Care was taken to obtain a good visualization of all chambers and a reliable delineation of the endocardial border. The measurements from three consecutive heart cycles were recorded and averaged. The frame rate was 60–80 frames/sec. An analysis was performed offline by a single experienced and independent echocardiographer, who was not directly involved in the image acquisition and blinded to the basic echocardiographic parameters, using semi-automated 2D strain software (EchoPac, GE, Milwaukee, Wisconsin). The endocardial border was manually traced in the apical views, delineating a region of interest (ROI) at the lowest width of 6 segments for each view. Necessary manual adjustments of the ROI were then performed and the longitudinal strain curves for each segment were generated by the software. The LV global longitudinal strain (GLS) was calculated as the average of 4-, 2- and 3-chamber longitudinal strain curves. The global peak atrial longitudinal strain (PALS) and global peak atrial contraction strain (PACS) were calculated at the end of the atrial reservoir and contraction phase, respectively, as the average of all LA segments in the 4- and 2-chamber views using QRS as the starting point [23]. Care was taken not to foreshorten the LA and dedicated views were utilized for the LA analysis allowing a more reliable delineation of the atrial endocardial border. In patients in which an optimal visualization of the left atrium could not be guaranteed, the breath-hold technique was applied. 

### 2.5. Lung Function Tests

The following lung function measurements were recorded according to ATS/ERS standards [24,25] using a Jaeger body plethysmograph with corrections for temperature and barometric pressure: forced expiratory volume in the first second (FEV1), forced vital capacity (FVC), FEV1/FVC, total lung capacity (TLC), residual volume (RV), transfer factor of the lung for carbon monoxide (TLCO), alveolar volume (AV) and TLCO/AV. 

### 2.6. Statistical Analysis 

The data were expressed as median ± standard deviations unless otherwise reported. A normality data test was applied to the analysis of the study variables; for this issue, we used a Kolmogorov–Smirnov test and a D’Agostino–Pearson test. Comparisons between groups were performed by a t-test and one-way ANOVA (with a Dunn’s multiple comparison post-hoc test) with significance set at *p* ≤ 0.05. A correlation analysis was performed with the Pearson test. To compare the categorical variables, we used a Fisher’s exact test and a chi-squared test. Kaplan–Meyer curves were used to evaluate survival, major cardiac events and sarcoidosis worsening in the outcome analysis. Univariate and multivariate Cox regression (B: coefficient beta; HR: hazard ratio; HR > 1 indicates an increased risk of an event) were performed in order to investigate the correlations between the study outcomes (MACE and sarcoidosis relapses) and the variables (see Appendix A tables). The time to event endpoints were compared using a two-sided log-rank test. A Bland–Altman analysis was performed to evaluate the interobserver variability for the speckle tracking measures on 20 randomly selected patients analyzed by a second operator.

The statistical analysis, receiver operating characteristic (ROC) curves and graphic representations of the data were obtained using GraphPad Prism Version 5.0 software for Windows.

## 3. Results

### 3.1. Study Population

We retrospectively enrolled 172 patients in the study (111 females, 57.4 ± 12.6 years old); the demographic features, pulmonary functional parameters, clinical, laboratory and radiological data at the baseline are reported in Table 1. In line with the epidemiology of sarcoidosis, the majority of the study population were non-smoker females and the onset of the disease occurred more frequently in the fifth decade of life (median 49 years old). Extrapulmonary localizations of the disease were found in 56 patients (32.5%, 44 females); at the baseline, 99 patients (67 females) were on a steroid and/or an immunosuppressive therapy. No significant differences were found between the treated and non-treated patients in terms of sex or age prevalence and patients with an extrapulmonary disease were more frequently taking steroids at the moment of the inclusion in the study (OR 2.008, 95% CI 1.020–3.953, *p* = 0.0460).

On average, the respiratory functional assessment showed normal forced expiratory volumes but a mild impairment of DLCO was observed. 

In Table 2, we report the TTE measurements, including the STE parameters, collected from our population. There was an interobserver agreement of 98% for the overall LV-GLS measures and of 94% for the LA strain measures.

### 3.2. Outcome Analysis

Follow-up clinical data were available for the entire study population. On 1 October 2020, the median time of observation was 2217 days. During the follow-up, we observed 8 deaths (4.6%, 3 females, median of survival 1409 days), of which 3 were reported as death by a cardiovascular event. MACE, including death, were reported in 23 patients (16 females, median 1004 days from the baseline); these patients were significantly older (65 ± 11.1 vs. 57.5 ± 15.2 years old, *p* = 0.0022) and were also more frequently affected with an extrapulmonary disease and naïve from therapy although not reaching a statistical significance (*p* = 0.0865 and *p* = 0.1083, respectively). There were 4 cardiac-related hospitalizations (3 for acute heart failure, 1 for an acute coronary syndrome) and 7 major arrhythmias (4 atrial fibrillations, 1 sustained ventricular tachycardia and 1 ventricular fibrillation). No differences in terms of demographic features, clinical and respiratory functional parameters, heart disease risk factors, clinical phenotype of diseases or the presence of sarcoid lung fibrosis were found between the two subgroups. 

**Figure 1:**
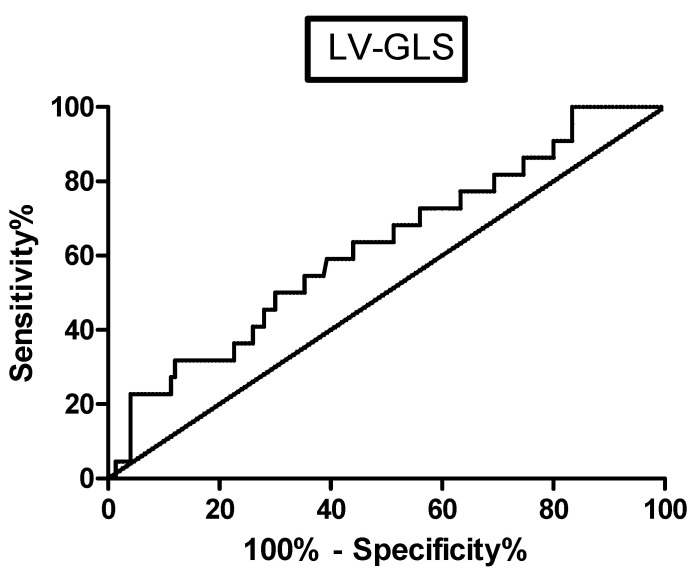
ROC curve for the evaluation accuracy of LV-GLS in predicting MACE.

**Figure 2:**
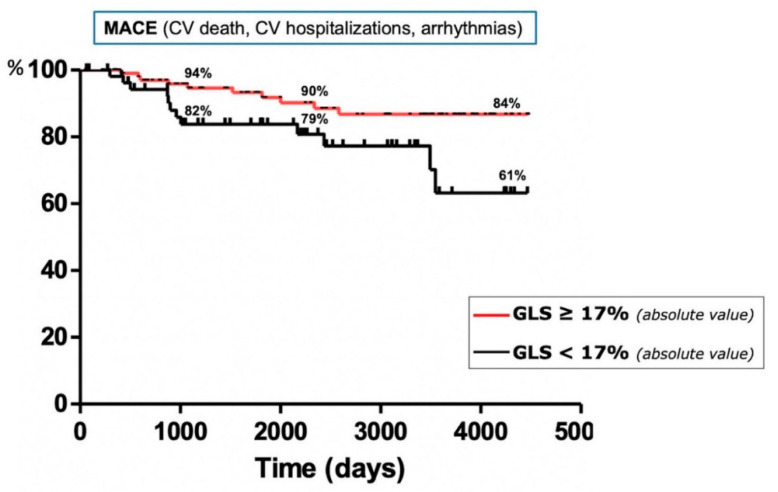
Kaplan–Meier curves designed for the MACE outcomes. The study population is stratified according to the cut-off value of the LV-GLS.

Concerning the echocardiographic parameters, the LV-GLS was significantly lower in patients with MACE (*p* = 0.0251); a ROC curve assessment confirmed the statistical significance of this finding (AUC 0.6420, *p* = 0.03177) (Figure 1), reporting a sensitivity of 50% and specificity of 70% for a cut-off value of 17.13 (likelihood ratio of 1.67). The Kaplan–Meier curves confirmed that sarcoidosis patients with GLS < 17.13% were associated with a higher rate of MACE (log-rank test 5.449, *p* = 0.0196) (Figure 2). No other baseline echocardiographic parameters showed significant differences between patients with or without MACE during the follow-up (Table 2). The univariate Cox analysis showed that an older age, lower serum ACE values and a decreased LV-GLS were significantly associated with MACE incidence in the follow-up (*p* = 0.002, *p* = 0.023 and *p* = 0.017, respectively) (Table A1); however, a multivariate analysis confirmed the statistical significance only for LV-GLS (HR 0.855, 95% CI 0.755–0.968, *p* = 0.013) (Table A2).

**Figure 3:**
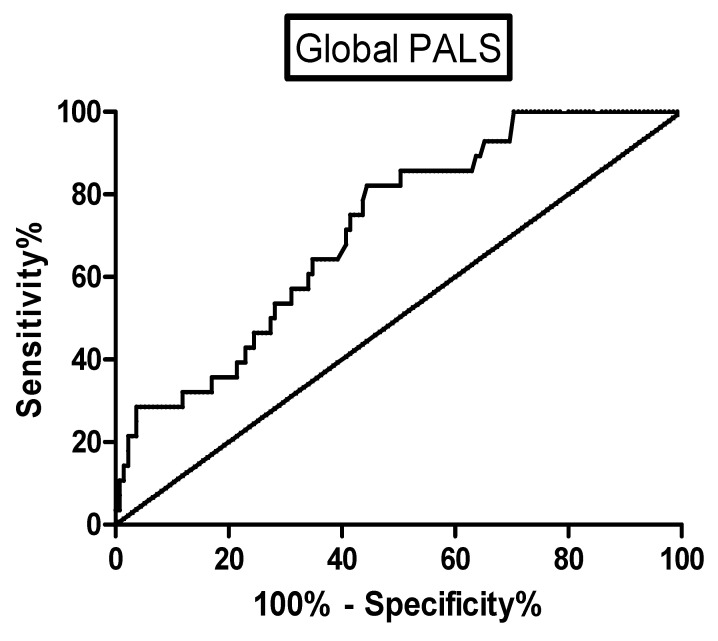
ROC curve for the assessment of the accuracy of global PALS in the prediction of a sarcoidosis relapse.

**Figure 4:**
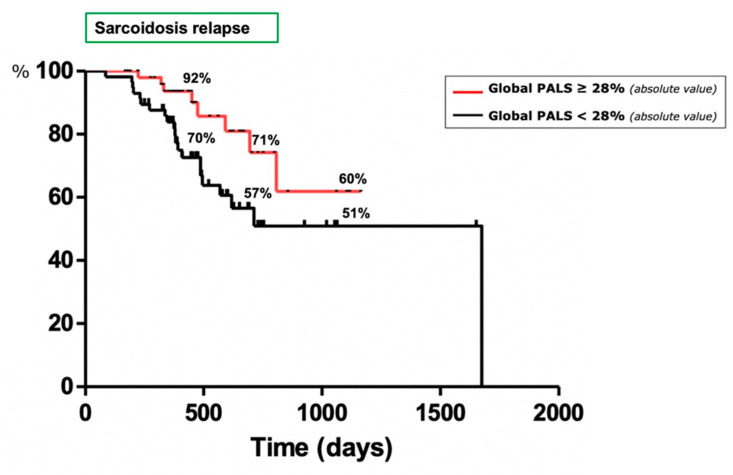
Kaplan–Meier curves designed for the outcome of sarcoidosis relapses. The study population is stratified according to the global PALS cut-off.

Regarding the control of sarcoidosis, 36 patients (24 females) showed a clinically relevant relapse of the disease during the follow-up (median time to event 428 days). We did not observe any significant differences of demographic, clinical, functional, radiological and therapeutic data between patients with a stable and worsening disease. An echocardiographic assessment revealed that patients with a relapse of sarcoidosis showed significantly lower values of EF%, TAPSE and global PALS (55.1 ± 10.2 vs. 57.8 ± 5.6 %, *p* = 0.0432; 19.7 ± 4.1 vs. 21.2 ± 3.3 mm, *p* = 0.0272; 25.6 ± 6.7 vs. 32 ± 8.2%, *p* = 0.0012, respectively) than patients with a stable disease during the follow-up. To compare the accuracy of these three indicators, ROC curves were plotted reporting the best performance for global PALS (AUC = 0.7155, *p* = 0.00034; sensitivity 64.3% and specificity 65.2% for a cut-off of 28.54) (Figure 3). Figure 4 shows the Kaplan–Meier curves of the study population stratified according to the cut-off of this indicator. We observed a higher probability of a sarcoidosis relapse in patients with a worse global PALS (log-rank test 4.411, *p* = 0.0357). The univariate Cox analysis confirmed that the global PALS values were significantly associated with a higher risk of a disease relapse (*p* < 0.001) as well as diabetes mellitus (*p* = 0.036) and global PACS (*p* = 0.003) (Table A3) but global PALS was the only parameter to show a significant impact on this outcome in the multivariate analysis (HR 12.736, 95% CI 1.850–87.664, *p* = 0.005) (Table A4).

## 4. Discussion

In the present study, we investigated the potential utility of TTE with an STE technique as a tool for the prognostic estimation of MACE and disease relapse in a large cohort of patients affected with sarcoidosis. In the last decade, TTE has gained leadership among cardiovascular imaging tools thanks to its non-invasive nature, reproducibility and zero emission of ionizing radiations [26,27]; however, the role of TTE in the management of sarcoidosis patients is still a matter of debate. Although sarcoidosis patients are associated with a higher risk of cardiovascular death or events than the general population [28,29], the effectiveness of traditional TTE as a prognostic tool in this setting is reported to be low. Moreover, in the diagnostic pathway of cardiac sarcoidosis, TTE is hindered by low sensitivity [30,31,32], which brings ATS to suggest cardiac MRI and PET as first-line imaging modalities for diagnostic and prognostic purposes in sarcoidosis [12]. However, recent evidence reported that the application of STE may significantly enhance the diagnostic and prognostic performance of TTE in the clinical management of sarcoidosis patients. On this topic, our results showed that the STE parameters—and GLS particularly—were more accurate in predicting MACE than traditional TTE parameters such as EF or PAPs, suggesting their implementation in the clinical practice for the risk stratification of these patients. Our data are in line with previous studies published on this setting both in patients with or without evidence of sarcoid cardiac involvement [14,16,33] and further supports the usefulness of GLS because our results were observed in a much larger cohort with a longer follow-up than previously reported. GLS is an emerging sensitive parameter of the LV function and has been accepted as a predictor of mortality in various clinical settings [34,35,36,37,38]; despite the low incidence of death and/or cardiac events in our cohort, GLS proved to be the most accurate parameter in the outcome analysis and, therefore, should be considered in the clinical assessment of sarcoidosis patients.

Our study focused on the potential impact of the STE parameters in the risk estimation of sarcoidosis relapses. To our knowledge, this is the first study to investigate this specific issue. Sarcoidosis is an extremely heterogeneous disease and the clinical course of disease as well as the response to treatment is still unpredictable at the moment of diagnosis. Therefore, new bioindicators able to assist the physician in the management of these patients are urgently needed. Our results showed that three parameters (EF, TAPSE and global PALS) were significantly lower at the baseline in patients experiencing a relapse of the disease in the follow-up. Among these parameters, despite being statistically significant, the difference of EF was too small to be considered clinically relevant. TAPSE is commonly used to assess the right ventricular function and a reduction of this parameter may suggest a more severe impairment of the pulmonary parenchyma and/or vasculature; however, in our population, we did not observe any correlation between TAPSE and respiratory functional parameters and pulmonary hypertension was diagnosed in only one patient. Moreover, the TAPSE levels in patients with a relapse—even if lower—remained, on average, in the normality range; as the TAPSE assessment is fully operator-sensitive, the clinical significance of this finding may be disputable. Finally, the global PALS showed quite surprisingly the best performance among the echocardiographic parameters as a predictor of a sarcoidosis relapse. Our results are intriguing and suggest that this parameter may be useful in the prognostic estimation of sarcoidosis patients. Global PALS is an emerging indicator of LA functionality and has been associated with the early detection of myocardial damage in arterial hypertension, diabetes mellitus and many other cardiological clinical settings [26,39,40]. To date, a left atrial assessment has been poorly investigated in sarcoidosis patients and it has been predominantly related to the onset of supraventricular rhythm disorders. From a mechanistic point of view, an impairment of the LA functionality is indicative of an early LV diastolic dysfunction, which has been associated with chronic inflammatory diseases such as rheumatoid arthritis (RA). As with RA, active sarcoidosis is characterized by an overexpression of many pro-inflammatory cytokines (e.g., tumor necrosis factor-α, interleukin-1 and inteleukin-6) that can induce a significant impairment of the left atrial and ventricular function [41,42]. Thus, our findings suggest that LA contractility may be early influenced by sarcoidosis inflammation, making global PALS a potential index of the activity of the disease and the risk of relapse.

Our study has a few limitations. First of all is the retrospective and monocenter nature of the study, which is typically prone to referral and reporting bias that may influence the collection and analysis of the data. Second, first-line cardiac imaging modalities such as cardiac MRI and PET were available only for four patients so we were not able to compare TTE and MRI/PET findings.

## 5. Conclusions

In conclusion, our study supports the utility of TTE-STE in the clinical management of sarcoidosis patients regardless of the evidence or the suspicion of cardiac localizations of the disease. Specific echocardiographic parameters showed promising results in predicting MACE and the risk of a sarcoidosis relapse. These findings need to be further validated in large, prospective and multicentric studies. 

## Figures and Tables

**Table 1:** Demographic, clinical, biochemical, functional and radiologic characteristics of the study population. All data are expressed as mean ± standard deviation (SD).

Parameters	Study Population	No MACE	MACE	Deaths
N°	172	149	23	8
Age (years)	57.4 ± 12.6	57.5 ± 15.2	65.1 ± 11.1	72.3 ± 5.6
Female sex (%)	111 (64.5)	95 (63.7)	16 (69.5)	3 (37.5)
Height (cm)	166 ± 10	165.8 ± 10.4	164.9 ± 6.1	164.7 ± 7.5
Weight (kg)	74 ± 16	74.3 ± 16.4	70.4 ± 12	76 ± 13.9
Body surface area (BSA) (m²)	1.8 ± 0.3	1.7 ± 0.3	1.7 ± 0.1	1.8 ± 0.2
Smokers n (%)	47 (27.3)	42 (28.1)	5 (21.7)	3 (37.5)
- Current	7 (4)	7 (4.6)	0 (0)	0 (0)
- Former	40 (23.2)	35 (23.4)	5 (21.7)	3 (37.5)
Patients with ongoing therapy	86 (50)	76 (51)	10 (43.4)	4 (50)
Time from diagnosis (mo)	45.6 ± 33.4	43.2 ± 28.6	55.4.7 ± 30.5	74.2 ± 85.5
*Clinical features*				
Hypertension (%)	42 (24.4)	34 (22.8)	8 (34.7)	2 (25)
Diabetes (%)	13 (7.5)	10 (6.7)	3 (13)	2 (25)
Ischemic heart disease (%)	25 (14.5)	17 (11.4)	8 (34.7)	2 (25)
Osteopenia/osteoporosis (%)	57 (33.1)	53 (35.5)	4 (17.4)	3 (37.5)
Psychiatric disorders (%)	11 (6.3)	9 (6)	2 (8.6)	0 (0)
GERD (%)	35 (20.3)	28 (18.8)	7 (30.4)	2 (25)
Other (%)	27 (15.6)	20 (13.4)	7 (30.4)	4 (50)
**Clinical phenotypes of sarcoidosis**				
Löfgren syndrome (%)	13 (7.5)	12 (8)	1 (4.3)	0 (0)
Extrapulmonary disease (%)	56 (32.5)	45 (30.2)	11 (47.8)	3 (37.5)
GenPhenResA classification				
- Abdominal (%)	10 (5.8)	10 (6.7)	0 (0)	0 (0)
- OCCC (%)	8 (4.6)	6 (4)	2 (8.6)	1 (12.5)
- Musculoskeletal cutaneous (%)	27 (15.6)	21 (14.1)	6 (26.1)	1 (12.5)
- Isolated pulmonary (%)	116 (67.4)	102 (68.4)	14 (60.8)	6 (75)
- Extrapulmonary (%)	11 (6.3)	10 (6.7)	1 (4.3)	0
**COS scale**				
1 (%)	9 (5.2)	8 (5.3)	1 (4.3)	0 (0)
2 (%)	15 (8.7)	14 (9.3)	1 (4.3)	0 (0)
3 (%)	13 (7.5)	11 (7.3)	2 (8.6)	0 (0)
4 (%)	8 (4.6)	7 (4.6)	1 (4.3)	1 (12.5)
5 (%)	7 (4)	5 (3.3)	2 (8.6)	1 (12.5)
6 (%)	13 (7.5)	11 (7.3)	2 (8.6)	0 (0)
7 (%)	38 (22.1)	34 (22.8)	4 (17.3)	2 (25)
8 (%)	51 (29.6)	45 (30.2)	6 (26.1)	2 (25)
9 (%)	18 (10.4)	14 (9.3)	4 (17.3)	2 (25)
Chest X-ray staging	156 (90.6)	133 (89.2)	23 (100)	8 (100)
Stage 0 n (%)	67 (42.9)	57 (38.2)	10 (43.4)	5 (62.5)
Stage 1 n (%)	14 (8.9)	12 (8)	2 (8.6)	0 (0)
Stage 2 n (%)	23 (14.7)	18 (12)	5 (21.7)	0 (0)
Stage 3 n (%)	37 (23.7)	32 (21.4)	5 (21.7)	2 (25)
Stage 4 n (%)	15 (9.6)	14 (9.3)	1 (4.3)	1 (12.5)
Pulmonary function tests				
FVC (mL)	3391 ± 1132	3438 ± 1168	3119 ± 868	2606 ± 479
% FVC predicted	103 ± 19	103.5 ± 19	103.5 ± 21.6	86.5 ± 22.1
FEV1 (mL)	2585 ± 966	2632 ± 997	2328 ± 719	1878 ± 445
% FEV1 predicted	95.6 ± 20	96 ± 19.7	93 ± 19.6	77.7 ± 22.5
FEV1/FVC (%)	76 ± 7.5	76.2 ± 7.6	73.3 ± 5.6	70.2 ± 7.4
DLCO predicted (%)	78 ± 17	77.2 ± 16.6	80.6 ± 17.1	77.9 ± 21.6
Urine calcium over 24 h (mg/24 h)				

COS: clinical outcome status; DLCO: diffusing lung capacity for carbon monoxide; FEV1: forced expiratory volume in the 1st second; FVC: forced vital capacity.

**Table 2:** Basic and advanced echocardiographic characteristics of the study population stratified according to MACE occurrence and death during the observation time.

Parameters	Study Population	No MACE	MACE	Deaths	*p*-Value
N°	172	149	23	8	
LV end-diastolic volume (mL)	96.6 ± 32.6	96.6 ± 32.7	97 ± 33.1	101 ± 27.7	0.9622
LV end-systolic volume (mL)	40.8 ± 16	40.8 ± 16.1	41.2 ± 14.9	44.2 ± 16.9	0.8946
LV ejection fraction (%)	57.3 ± 6.7	57.5 ± 6.5	55.8 ± 7.5	56.8 ± 8	0.2730
LV mass index (g/m²)	86.9 ± 29	86.1 ± 26.2	92.1 ± 44.5	97.1 ± 58.8	0.5444
Left atrial area (cm²)	17.1 ± 4.2	17.1 ± 4.3	17.2 ± 4	18.4 ± 5.1	0.9082
Left atrial volume index (mL/m²)	27.4 ± 9.6	27.2 ± 9.8	28.7 ± 10.4	30 ± 13.7	0.5314
Mitral regurgitation ≥ moderate n (%)	37 (21.51)	34 (22.8)	3 (13)	2 (25)	0.4154
Tricuspid regurgitation ≥ moderate n (%)	31 (18.02)	29 (19.4)	2 (8.6)	2 (25)	0.2591
Right atrial area (cm²)	12.7 ± 3.3	12.6 ± 3.3	13.2 ± 3.5	13.5 ± 4.8	0.4661
TDI tricuspid wave (m/sec)	0.13 ± 0.03	0.13 ± 0.031	0.13 ± 0.028	0.14 ± 0.025	0.6082
TAPSE (mm)	20.9 ± 3.5	21 ± 3.5	20.8 ± 3.1	20.8 ± 3.6	0.8047
sPAP (mm/Hg)	29.4 ± 8.3	29.5 ± 8.6	28.7 ± 5.3	28.7 ± 5.3	0.7048
RV medium end-diastolic diameter (mm)	28.1 ± 5.1	28.3 ± 5.3	27.1 ± 4.1	27.2 ± 3.9	0.2148
RV sphericity index	0.62 ± 0.09	0.62 ± 0.11	0.61 ± 0.1	0.60 ± 0.2	0.6018
RV/LV ratio	0.56 ± 0.07	0.56 ± 0.08	0.54 ± 0.06	0.53 ± 0.08	0.1955
RVFAC (%)	42.9 ± 8.7	43 ± 8.4	42.6 ± 6.3	40.8 ± 7.3	0.8267
LV-GLS (%)	18.3 ± 3.6	18.5 ± 3.5	16.8 ± 3.7	14.6 ± 3.7	0.0251
Global PALS (%)	30.5 ± 9	30.8 ± 8.1	28.5 ± 10.5	26.9 ± 9.2	0.2700
Global PACS (%)	16 ± 12.7	16.1 ± 13.4	15.2 ± 5	15.0 ± 4.8	0.5834
PALS-4ch	30 ± 9.6	30.4 ± 9.6	27.6 ± 9.8	26.2 ±10.3	0.1969
PACS-4ch	14.6 ± 5.4	14.5 ± 5.3	15 ± 6.4	13 ± 5.4	0.6741
PALS-2ch	31.2 ± 9.4	31.5 ± 9.7	28.9 ± 8.4	27.7 ± 9.1	0.1554
PACS-2ch	17.6 ± 23.7	17.9 ± 25.3	15.4 ± 5	14.8 ± 6.3	0.6389
LS-4ch	18.1 ± 3.6	18.3 ± 3.6	17.8 ± 3.1	17.5 ± 2.7	0.1638
LS-2ch	18.7 ± 4.8	19.2 ± 3.8	17.3 ± 8.1	15.9 ± 11.9	0.0986
LS-3ch	18.1 ± 3.7	18.3 ± 3.7	17 ± 3.3	16 ± 3.1	0.0619
Global RVLS	19.5 ± 4.1	19.6 ± 4.2	18.9 ± 2.8	17.6 ± 2.1	0.2084
Free wall RVLS	21.2 ± 5.2	21.4 ± 5.3	20.6 ± 3.8	20.2 ± 3.7	0.3661
PALS-4ch RA	34.1 ± 9.6	34.6 ± 9.6	31.8 ± 8.7	30.2 ± 12.1	0.0899
PACS-4ch RA	15.3 ± 5.4	15.4 ± 5.4	14.6 ± 5.8	15.2 ± 9.1	0.5379

GLS: global longitudinal strain; LV: left ventricle; PACS: peak atrial contraction strain; PALS: peak atrial longitudinal strain; RVLS: right ventricle longitudinal strain; RA: right atrium; RVFAC: right ventricular fractional area change; sPAP: systolic pulmonary artery pressure; TAPSE: tricuspid annular plane systolic excursion; TDI: tissue doppler imaging.

## Data Availability

The data presented in this study are available in Results’ Section.

## Appendix A

**Table A1:** Univariate Cox analysis of the study population for MACE.

	B	HR	CI (95%)	*p*-Value
Baseline Features				
Age	0.063	1.065	1.023–1.108	**0.002**
Sex	−0.370	0.691	0.269–1.775	0.442
Hypertension	−0.426	0.653	0.273–1.562	0.338
Former smoke habit	−0.322	0.725	0.245–2.144	0.561
Current smoke habit	3.079	21.731	0.003–156.170	0.497
Diabetes mellitus	−0.234	0.791	0.185–3.391	0.752
ACE	−0.023	0.977	0.956–0.998	**0.032**
Lysozyme	0.122	1.130	0.903–1.413	0.286
Chitotriosidase	−0.001	0.999	0.996–1.002	0.415
Urinary calcium	−0.001	0.999	0.994–1.005	0.801
Löfgren syndrome	0.528	1.696	0.220–13.058	0.612
Extrapulmonary localizations	−0.374	0.688	0.297–1.593	0.383
X-ray stage of disease	−0.095	0.909	0.679–1.218	0.522
Therapy	0.530	1.699	0.725–3.984	0.223
Spirometry data				
FVC% predicted value	−0.001	0.999	0.978–1.021	0.922
FVC (mL)	0.001	1.001	0.999–1.001	0.479
FEV1 % predicted value	−0.007	0.993	0.972–1.014	0.487
FEV1 (mL)	0.001	1.001	0.999–1.001	0.427
FEV1/FVC	−0.032	0.969	0.924–1.015	0.184
DLCO % predicted value	0.008	1.008	0.983–1.035	0.526
Echocardiographic data				
IVS	−0.042	0.959	0.822–1.119	0.596
LV-EDD	0.059	1.060	0.982–1.145	0.135
EDV	−0.003	0.997	0.984–1.010	0.620
LVEF	−0.021	0.979	0.924–1.037	0.474
Indexed LA volume	0.023	1.023	0.985–1.063	0.233
E/E′	−1.698	0.183	0.034–0.990	0.149
Deceleration time	0.003	1.003	0.996–1.010	0.359
Mitral regurgitation	−0.596	0.551	0.206–1.472	0.235
Right mid-ventricular EDD	−0.016	0.984	0.891–1.087	0.756
RA area	0.110	1.116	0.994–1.252	0.063
TAPSE	0.017	1.017	0.894–1.156	0.800
Tricuspid regurgitation	−0.253	0.777	0.426–1.417	0.410
sPAP	−0.005	0.995	0.931–1.063	0.885
Strain echocardiographic data				
Global LA PALS	−0.050	0.952	0.907–0.998	0.063
Global LA PACS	−0.026	0.974	0.894–1.061	0.551
LV-GLS	−0.141	0.869	0.774–0.975	**0.017**
TTP LV-GLS	0.005	1.005	1.000–1.010	0.068
RV-GLS	−0.036	0.965	0.852–1.093	0.574
TTP RV-GLS	−0.002	0.998	0.991–1.005	0.555
Free wall RVLS	−0.012	0.988	0.904–1.079	0.782

**Table A2:** Multivariate Cox analysis of the study population for MACE.

	B	HR	CI (95%)	*p*-Value
Baseline Features				
Age	0.001	1.001	1.000–1.003	0.664
Female sex	−0.053	0.948	0.341–2.634	0.918
ACE	−0.014	0.986	0.966–1.007	0.195
X-ray stage of disease	−0.094	0.910	0.658–1.258	0.568
Global LA PALS	0.080	1.083	0.940–1.248	0.270
LV-GLS	−0.156	0.855	0.756–0.968	**0.013**

**Table A3:** Univariate Cox analysis of the study population for a sarcoidosis relapse.

	B	HR	CI (95%)	*p*-Value
Baseline Features				
Age	0.001	1.001	1.001–1.006	0.955
Sex	−0.073	0.929	0.464–1.863	0.836
Hypertension	0.264	1.302	0.592–2.862	0.511
Former smoke habit	0.068	1.071	0.488–2.352	0.865
Current smoke habit	3.079	21.728	0.023–209.123	0.380
Diabetes mellitus	−0.941	0.390	0.162–0.941	**0.036**
ACE	0.011	1.011	0.996–1.027	0.151
Lysozyme	0.148	1.160	0.991–1.358	0.064
Chitotriosidase	−0.001	0.999	0.998–1.001	0.473
Urinary calcium	0.001	1.001	0.996–1.004	0.894
Löfgren syndrome	0.324	1.383	0.328–5.833	0.659
Extrapulmonary localizations	0.057	1.058	0.524–2.137	0.875
X-ray stage of disease	0.085	1.089	0.869–1.365	0.459
Therapy	−0.135	0.873	0.436–1.748	0.702
Spirometry data				
Predicted FVC	0.001	1.001	0.983–1.018	0.999
FVC (mL)	0.001	1.001	1.002–1.004	0.623
FEV1 % predicted value	−0.004	0.996	0.979–1.014	0.680
FEV1 (mL)	0.002	1.002	1.001–1.005	0.589
FEV1/FVC	−0.006	0.994	0.954–1.037	0.786
DLCO % predicted value	−0.002	0.998	0.977–1.019	0.829
*Echocardiographic data*				
IVS	−0.022	0.978	0.885–1.081	0.669
LV-EDD	−0.031	0.970	0.940–1.000	0.051
EDV	−0.014	0.986	0.974–0.998	0.220
LVEF	−0.017	0.983	0.939–1.028	0.451
Indexed LA volume	0.005	1.005	0.973–1.038	0.774
E/E′	−0.081	0.922	0.376–2.261	0.859
Deceleration time	0.006	1.006	1.001–1.012	0.200
Mitral regurgitation	−0.676	0.509	0.234–1.105	0.088
Right mid-ventricular EDD	0.010	1.010	0.949–1.075	0.765
RA area	0.031	1.031	0.933–1.140	0.548
TAPSE	−0.052	0.950	0.862–1.045	0.292
Tricuspid regurgitation	−0.327	0.721	0.442–1.175	0.190
sPAP	0.013	1.013	0.974–1.053	0.525
Strain echocardiographic data				
Global LA PALS	−0.072	0.931	0.897–0.966	**<0.001**
Global LA PACS	−0.098	0.907	0.850–0.967	**0.003**
LV-GLS	−0.053	0.949	0.861–1.045	0.286
TTP LV-GLS	−0.004	0.996	0.992–1.001	0.093
RV-GLS	−0.051	0.951	0.866–1.043	0.284
TTP RV-GLS	−0.002	0.998	0.992–1.003	0.458
Free wall RVLS	−0.020	0.980	0.916–1.048	0.551

**Table A4:** Multivariate Cox analysis of the study population for a sarcoidosis relapse.

	B	HR	CI (95%)	*p*-Value
Diabetes Mellitus	−0.790	0.454	0.152–1.357	0.157
Age	0.005	1.002	1.000–1.008	0.985
Global LA PALS	2.544	12.736	1.850–87.664	0.005
Global LA PACS	0.149	1.161	0.880–1.531	0.291
